# *Lysinibacillus xylanilyticus* Strain GIC41 as a Potential Plant Biostimulant

**DOI:** 10.1264/jsme2.ME21047

**Published:** 2021-11-05

**Authors:** Nusrat Ahsan, Malek Marian, Haruhisa Suga, Masafumi Shimizu

**Affiliations:** 1 The United Graduate School of Agricultural Science, Gifu University, 1–1 Yanagido, Gifu, Gifu 501–1193, Japan; 2 Life Science Research Center, Gifu University, 1–1 Yanagido, Gifu, Gifu 501–1193, Japan

**Keywords:** *Lysinibacillus*, plant growth promotion, spinach, plant biostimulant

## Abstract

To identify *Lysinibacillus* strains with the potential to function as plant biostimulants, we screened 10 previously isolated *Lysinibacillus* strains from the rhizosphere and soil for their plant growth-promoting (PGP) effects. *In vitro* tests showed that all strains produced indole-3-acetic acid. In primary screening, the PGP effects of these strains were assessed on spinach seedlings grown on Jiffy-7 pellets; strains GIC31, GIC41, and GIC51 markedly promoted shoot growth. In secondary screening, the PGP efficacies of these three strains were examined using spinach seedlings grown in pots under controlled conditions. Only GIC41 exerted consistent and significant PGP effects; therefore, it was selected for subsequent experiments. The results of 6-week glasshouse experiments revealed that GIC41 markedly increased shoot dry weight by *ca.* 12–49% over that of the control. The impact of fertilization levels on the PGP efficacy of GIC41 was investigated using pot experiments. The application of a specific level of fertilizer was required for the induction of sufficient PGP effects by this strain. The phylogenetic ana­lysis based on the 16S rDNA sequence identified GIC41 as *L. xylanilyticus*. Collectively, these results show the potential of strain GIC41 to function as a plant biostimulant.

In agriculture, large amounts of chemical fertilizers are commonly used to increase crop production and meet the global food demands of the increasing population. Public concerns related to the impact of chemical fertilizers on the environment have also increased (Gupta *et al.*, 2015). The long-term and indiscriminate use of chemical fertilizers degrades soil quality and has been linked to a number of environmental issues (Savci, 2012). Moreover, the production process of fertilizers involves the utilization of large amounts of energy (accounting for 1.2% of the world’s gross energy needs) and the emission of carbon dioxide (CO_2_) and other greenhouse gasses (Woods *et al.*, 2010; Ghavam *et al.*, 2021). Therefore, the implementation of environmentally friendly and effective strategies that reduce dependency on chemical fertilizers is urgently needed. As one of the possible solutions to this issue, the use of plant biostimulants is gaining significant interest by scientific communities and agricultural industries based on their potential to enhance crop productivity in a sustainable manner (Rouphael and Colla, 2020; Hamid *et al.*, 2021). Plant biostimulants are substances or microorganisms that are applied to plants in order to enhance nutrition efficiency, abiotic stress tolerance, and/or crop quality traits regardless of their nutrient content (du Jardin, 2015). Among the active ingredients of plant biostimulants, plant growth-promoting bacteria (PGPB), living in or on soil, the rhizosphere, and plant tissues, have been attracting increasing attention. PGPB promote plant growth through a diverse range of direct and indirect mechanisms (Glick, 2012; Olanrewaju *et al.*, 2017). Bacteria of diverse genera, such as *Azospirillum*, *Bacillus*, *Burkholderia*, *Paenibacillus*, *Pseudomonas*, and *Rhizobia*, have been identified as PGPB and extensively examined in an attempt to develop plant biostimulants for a number of crops (Govindasamy *et al.*, 2010; Suárez-Moreno *et al.*, 2012; Panpatte *et al.*, 2016; Cassán *et al.*, 2020; Lindström and Mousavi, 2020). Among the PGPB group, *Bacillus* species are one of the most common and effective ingredients of plant biostimulants (Nguyen *et al.*, 2019) because this genus produces endospores that are highly resistant to a wide variety of abiotic environmental stresses, such as dryness, UV radiation, and high temperature, and the preparation of commercial formulations of plant biostim­ulants is easier and inexpensive. To enrich the portfolio of microbe-based plant biostimulants, it is essential to search for these types of practical PGPB.

*Lysinibacillus* is a newly classified genus that was previously classified as *Bacillus* (Ahmed *et al.*, 2007). Since *Lysinibacillus* species also have a demonstrated ability to form endospores, they are expected to serve as suitable agents for microbe-based products, including plant biostimulants (Ahsan and Shimizu, 2021). *L. sphaericus*, a well-known entomopathogen, has been formulated as mosquito insecticides that are commercially available worldwide (Berry, 2012). Recent studies demonstrated that the *Lysinibacillus* species are ubiquitous in nature, may be isolated from the plant rhizosphere, phyllosphere, and from inside plant tissues, and possess plant growth-promoting (PGP) traits, such as auxin production, phosphate solubilization, siderophore production, and nitrogen fixation (Vendan *et al.*, 2010; Trivedi *et al.*, 2011; Sharma and Saharan, 2015; Verma *et al.*, 2016). Moreover, some *Lysinibacillus* strains have been shown to promote plant growth (Sahu *et al.*, 2018; Shabanamol *et al.*, 2018).

Our laboratory has a collection of various types of bacteria isolated from the rhizosphere, plant tissues, and soil, including strains of the genus *Lysinibacillus*. In the present study, we examined the PGP activities of our *Lysinibacillus* strains with the aim of selecting potential plant biostimulants.

## Materials and Methods

### Bacterial strains

The *Lysinibacillus* strains used in the present study were selected from our laboratory’s bacterial collection based on their partial 16S rRNA gene sequences. They are listed in Table 1. Strains GIC31, GIC41, GIC51, GIC81, and GIC119 were originally isolated from paddy field soil, whereas strain GUCS34 was isolated from tea garden soil. Strains T16 and T20 were derived from the rhizosphere soil of tomato (*Solanum lycopersicum* L.). The remaining two strains, C75 and W30, were isolated from the rhizosphere soil of Chinese chive (*Allium tuberosum* Rottler ex Spreng.) and Welsh onion (*Allium fistulosum* L.), respectively.

Full-length 16S rRNA gene sequences were elucidated as suggested by Fu *et al.* (2020) for a more precise taxonomic classification. The 16S rRNA gene sequences of the strains were submitted to the GenBank database. These sequences were analyzed using a global alignment algorithm implemented in the EzBioCloud database (https://www.ezbiocloud.net/) (Yoon *et al.*, 2017) and compared with the sequences of known *Lysinibacillus* species in the same database. A phylogenetic tree was constructed with the neighbor-joining method using MEGA version 7.24.2827 (Kumar *et al.*, 2018).

### Inoculum preparation

*Lysinibacillus* strains were cultured on nutrient broth (Nissui Pharmaceutical) at 30°C for 2 days with shaking at 200‍ ‍rpm. Cells were harvested via centrifugation at 9,900×*g* for 10‍ ‍min and washed twice with 10‍ ‍mM MgCl_2_·6H_2_O. Washed cells were resuspended in 10‍ ‍mM MgCl_2_·6H_2_O and adjusted to an optical density at 600‍ ‍nm (OD_600_) of 0.5 (*ca.* 10^7^ colony-forming units [CFU] mL^–1^).

### Assessment of indole-3-acetic acid (IAA) production

The abilities of bacterial strains to produce IAA were examined by the method suggested by Kumar *et al.* (2012) with some modifications. In brief, a 30-μL aliquot of the cell suspension of each strain was inoculated into 10‍ ‍mL of tryptic soy broth (TSB; Becton, Dickinson and Company) supplemented with 0.5‍ ‍mg of L-tryptophan and incubated with shaking at 200‍ ‍rpm at 30°C for 2 days. After the incubation, the culture broth was centrifuged at 5,000×*g* for 10‍ ‍min. The supernatant (2‍ ‍mL) was carefully pipetted out and mixed with 2‍ ‍mL of Salkowski reagent (1‍ ‍mL of 0.5 M FeCl_3_ solution in 50‍ ‍mL of 35% perchloric acid) (Gordon and Weber, 1951). IAA concentrations were measured colorimetrically by comparing the OD_530_ value of standard pure IAA with the culture supernatant of each bacterial strain.

### Assessment of phosphate-solubilizing abilities

The cell suspension of each *Lysinibacillus* strain was spotted onto Pikovskaya’s agar medium supplemented with tricalcium phosphate (Pikovskaya, 1948) and incubated at 30°C for 14 days. The phosphate-solubilizing abilities of the bacterial strains were indicated by the appearance of a clear zone around bacterial colonies. In this experiment, *Flavobacterium* sp. strain GFA4, which was previously identified as a phosphate-solubilizing bacterium, was cultured on the same medium as the positive control and it was confirmed that this strain produced a clear zone around its colony.

### Siderophore production assay

The production of siderophores by bacterial strains was assessed using the overlaid chrome azurol S agar (O-CAS) assay (Pérez-Miranda *et al.*, 2007) with some modifications. In brief, the cell suspension of each bacterial strain was spotted onto iron-free King’s B medium and incubated at 30°C. After 1‍ ‍week of incubation, chrome azurol S (CAS) agarose (Schwyn and Neilands, 1987) was applied to King’s B plates and incubated for 30‍ ‍min. The development of a yellow/orange halo in overlaid agarose around the bacterial colonies was considered to be positive for siderophore production.

### Assessment of 1-aminocyclopropane-1-carboxylate (ACC) deaminase activity

The activity of ACC deaminase was evaluated according to the method described by Dell’Amico *et al.* (2005). In brief, bacterial strains were grown in TSB. After centrifugation, a 30-μL aliquot of the cell suspension (OD_600_=0.5) of each strain was transferred to Dworkin and Faster (DF) medium containing 3.0‍ ‍mM ACC as the sole nitrogen source and incubated with shaking at 200‍ ‍rpm at 30°C for 2 days. DF medium without any nitrogen source was used as the control. Bacterial growth was assessed by measuring optical density at 600‍ ‍nm.

### Assessment of nitrogen-fixing potential

The nitrogen-fixing potential of bacterial strains was assessed by the method of Setten *et al.* (2013) with some modifications. Bacterial strains were grown for 24 h in L medium (Setten *et al.*, 2013) containing 7.57‍ ‍mM (NH_4_)_2_SO_4_ at 30°C with shaking at 200‍ ‍rpm. After the incubation, bacterial cells were harvested by centrifugation at 5,000×*g* for 10‍ ‍min and washed twice with nitrogen-free L medium. Washed cells were resuspended in nitrogen-free L medium and adjusted to OD_600_ of 0.5. A 50-μL aliquot of the cell suspension was then inoculated into 5‍ ‍mL of nitrogen-free L medium in a 15-mL tube and incubated at 30°C with shaking at 200‍ ‍rpm under aerobic (test tube covered with parafilm) and micro-aerobic (test tube with plastic screw caps) conditions. After 48 h of incubation, bacterial cell density (OD_600_) was measured using a spectrophotometer.

### Primary screening for PGP strains

The effects of the *Lysinibacillus* inoculation on the growth of spinach was evaluated to identify potential PGP strains. Spinach seeds (*Spinacia oleracea* L. cv. Banchu-summer-sky) were surface sterilized with 70% (v/v) ethanol for 1‍ ‍min, followed by a treatment with 2% sodium hypochlorite for 5‍ ‍min and thorough rinsing with sterilized distilled water. Seeds were then placed on moist filter paper in a Petri dish and vernalized at 4°C for 1 day. Thereafter, seeds were sown on Jiffy-7 pellets (Jiffy Products International) covered with a small amount of sterilized vermiculite and grown in a controlled environmental chamber (Biotron LH-220S, Standard; Nippon Medical and Chemical Instruments) at 23°C under a 12-h light/12-h dark cycle (light intensity, 30,000‍ ‍Lx). After 7 days of sowing, spinach seedlings were drenched with 3‍ ‍mL of the bacterial cell suspension (OD_600_=0.5). Control plants were treated with an equal volume of sterile MgCl_2_·6H_2_O (10‍ ‍mM) without bacteria. Control and bacteria-treated seedlings were both maintained in the same controlled environmental chamber (light intensity, 30,000‍ ‍Lx). Plants were regularly irrigated throughout the growing period. After 20 days of the drench treatment, spinach plants were harvested from pellets, and their leaf areas and shoot dry weights were measured. Image ana­lysis software (LIA for Win32, https://www.agr.nagoya-u.ac.jp/~shinkan/LIA32) was used to measure the total leaf area of spinach plants from scanned images of all harvested leaves. To measure the shoot dry weight, spinach shoots (cut from the root and shoot junctions) were stored in a ventilated constant temperature dryer (ADVANTEC^®^ DRM620TB; Toyo Seisakusho) at 80°C for 2 days. In this experiment, five plants were used for each treatment, and the experiment was repeated three times. Differences in the leaf area and shoot dry weight between the control and bacterial treatments were analyzed by Dunnett’s test (*P*<0.05).

### Secondary screening for PGP strains

Strains GIC31, GIC41, and GIC51, the three best performing strains in primary screening, were subjected to further assessments in a pot experiment for their effects on spinach growth. Spinach seeds (cv. Banchu-summer-sky) were surface sterilized and vernalized as described above. Seeds were then sown in 150-mL plastic pots containing a double-autoclaved mixture of river sand and vermiculite at a ratio of 1:1 (v/v) and grown in a controlled environmental chamber at 23°C under a 12-h light/12-h dark cycle. After 7 days of sowing, seedlings were drenched with 10‍ ‍mL of the cell suspension of each strain (OD_600_=0.5). Control plants were drenched with the same amount of sterile MgCl_2_·6H_2_O (10‍ ‍mM). Plants were regularly irrigated and fertilized once a week with 0.2% (the concentration recommended by the supplier) Hyponex solution (Type: 6–10–5; Hyponex Japan) until excess solution leached out of the drainage holes in the bottoms of the pots. The first fertilization was performed on the day of the drench treatment. After 20 days of the drench treatment, plants were harvested, and their leaf areas and shoot dry weights were measured as described above. Ten plants were used for each treatment, and the experiment was repeated three times. Differences in the leaf area and shoot dry weight among the treatments were analyzed by Tukey’s test (*P*<0.05).

### Evaluation of PGP effects of strain GIC41 under glasshouse conditions

Strain GIC41 was selected as a potential PGP candidate because it performed the best in secondary screening. In this experiment, the PGP effects of this strain were evaluated under glasshouse conditions. Surface-sterilized and vernalized spinach seeds (cv. Banchu-summer-sky) were sown in plastic trays (Bee pot Y-49; Canelon Kaka) containing the commercial potting soil mix “Saika-ichiban” (IBIKO Corporation) and grown in a controlled environmental chamber at 23°C under a 12-h light/12-h dark cycle. After 7 days of sowing, seedlings were treated by drenching with the cell suspension of GIC41 (OD_600_=0.5) at 10‍ ‍mL per plant. Control seedlings were treated with sterile MgCl_2_·6H_2_O (10‍ ‍mM) instead of the GIC41 cell suspension. These seedlings were then transplanted into rectangular plastic containers (64×22×20‍ ‍cm) containing the commercial potting soil mix (five seedlings per container) and grown on a bench in a glasshouse under natural sunlight for 6‍ ‍weeks; during this period, plants were regularly irrigated without fertilization. Five containers were used as replicates for each treatment, and the experiment was repeated three times (designated as trials 1 to 3). Trials 1, 2, and 3 were conducted between March 26 and May 7, April 5 and May 17, and April 16 and May 28 in 2019, respectively.

At the end of the experiment in each trial, spinach plants were harvested and the shoot dry weight (after drying at 80°C for 2 days) was measured. The nitrogen and carbon contents of the shoots were measured using the dry combustion method with an NC analyzer (SUMIGRAPH NC TR22; Sumika Chemical Analysis Service). To conduct this experiment, dried spinach shoots were ground in a blender to prepare homogenized samples. Thereafter, the nitrogen and carbon contents of the powder were assessed using the NC analyzer device. Since the growth of spinach plants in the control treatment markedly varied among the trials, shoot dry weight data obtained from the three trials were subjected to a fixed effects meta-ana­lysis using the R package “meta” (ver. 2.6-1) (Schwarzer *et al.*, 2015). Differences in nitrogen and carbon content between the control and GIC41 treatment were analyzed by the Student’s *t*-test (*P*<0.05).

### Evaluation of PGP effects of GIC41 at different fertilization levels

In this experiment, the PGP effects of strain GIC41 were assessed at different fertilization levels. The experimental set-up was identical to that of the second screening experiment, except for the growth condition and fertilizer concentration. The experiment was conducted in a controlled environmental chamber (MLR-350; Sanyo Electric) at 23°C under a 12-h light/12-h dark cycle (light intensity, 20,000 Lx), and three different concentrations (0.05, 0.1, and 0.2%) of Hyponex solutions were provided to the spinach plants. After 20 days of drenching with the GIC41 cell suspension or sterile MgCl_2_·6H_2_O (10‍ ‍mM), spinach plants were uprooted from the pots, and their shoot and root dry weights and leaf areas were measured. Five plants were used for each treatment, and the experiment was repeated three times. Differences in leaf areas and shoot and root dry weights between the control and bacterial treatment were analyzed by Tukey’s test (*P*<0.05).

### Statistical ana­lysis

All statistical analyses were performed with EZR version 1.41 (Saitama Medical Center, http://www.jichi.ac.jp/saitama-sct/SaitamaHP.files/statmedEN.html), which is a graphical user interface for R (The R Foundation for Statistical Computing, version 3.6.1).

### Nucleotide sequence accession numbers

The nucleotide sequences of the full-length 16S rRNA genes were deposited in the GenBank database under accession numbers MZ520561, MZ543950, MZ543951, MZ543965, MZ543967, MZ543966, MZ543969, MZ543964, MZ543970, and MZ543972 (Table 1).

## Results

### Molecular characterization of *Lysinibacillus* strains

**Fig. 1.**
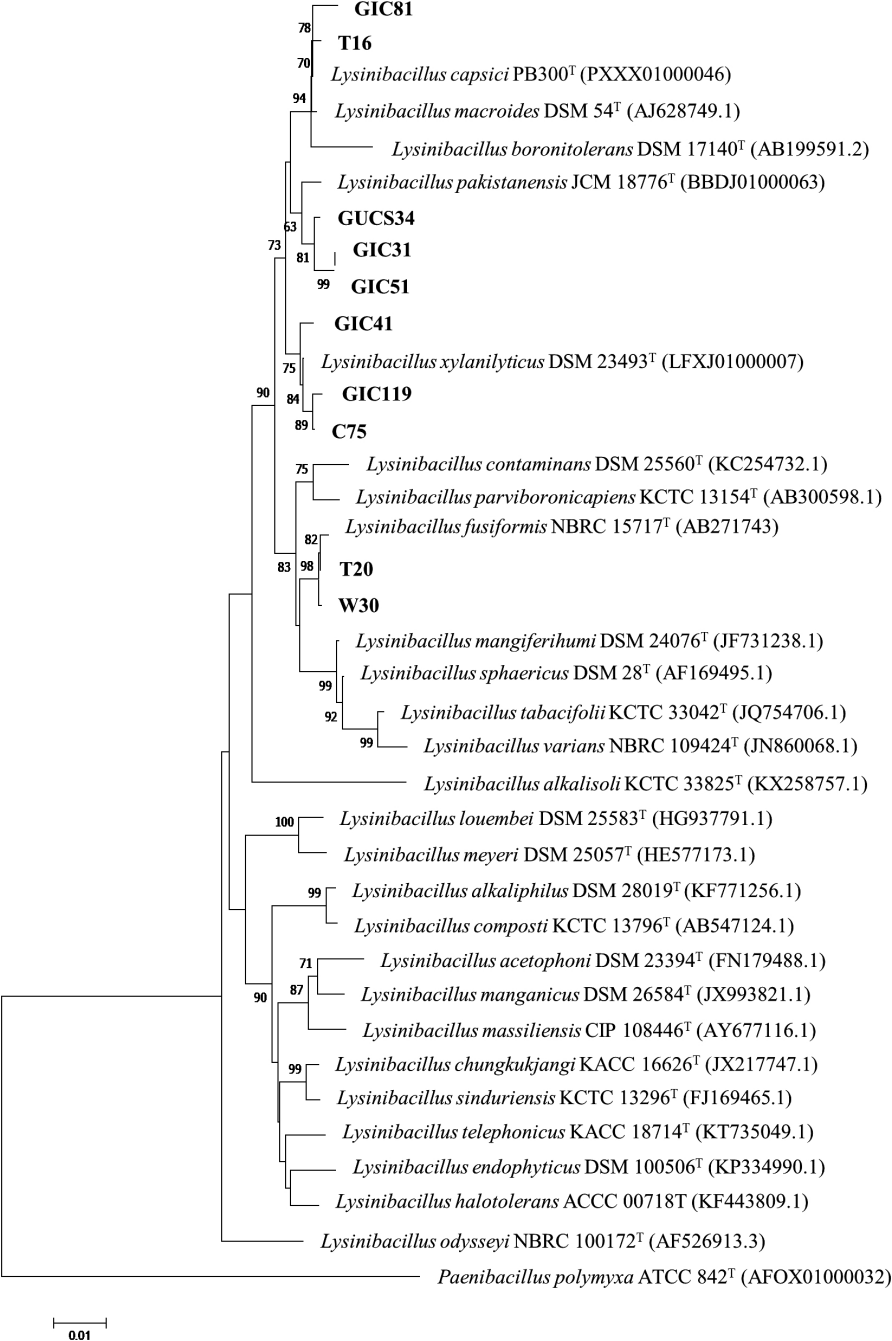
Phylogenetic positions of *Lysinibacillus* strains based on a complete 16S rRNA gene sequence ana­lysis. Bootstrap values of 1,000 replicates are shown next to the branches based on a neighbor-joining ana­lysis.

The 10 *Lysinibacillus* strains used in the present study were identified by a sequence ana­lysis of the nearly full-length 16S rRNA genes. The results obtained revealed that these strains possessed high similarities to species of *L. xylanilyticus*, *L. pakistanensis*, *L. capsici*, and *L. fusiformis* (Table 1). To clarify the phylogenetic positions of these strains, a phylogenetic tree was constructed based on the 16S rRNA gene sequences of these strains and the recognized type strains of *Lysinibacillus* species (Fig. 1). Strains GIC41, GIC119, and C75 formed a cluster with *L. xylanilyticus* DSM 23493^T^. Strains GIC31, GIC51, and GUCS34 were positioned in a cluster with *L. pakistanensis* JCM 18776^T^. Strains T20 and W30 formed a single cluster along with the most closely related strain of *L. fusiformis* NBRC 15717^T^. In contrast, the two remaining strains GIC81 and T16 were placed in a cluster that included *L. capsici* PB 300^T^, *L. macroides* DSM 54^T^, and *L. boronitolerans* DSM 17140^T^.

### *In vitro* PGP traits

*Lysinibacillus* strains were tested *in vitro* for their abilities to produce IAA, siderophores, and ACC deaminase, solubilize phosphate, and fix nitrogen. All strains were capable of producing IAA, but lacked the other traits (Table 2). Among the strains tested, IAA production was the highest in C75 at 2.0‍ ‍μg mL^–1^ of IAA, followed by T20 and GUCS34 at 1.9 and 1.8‍ ‍μg mL^–1^ of IAA, respectively.

### Screening for effective PGP *Lysinibacillus* strains in spinach

**Fig. 2.**
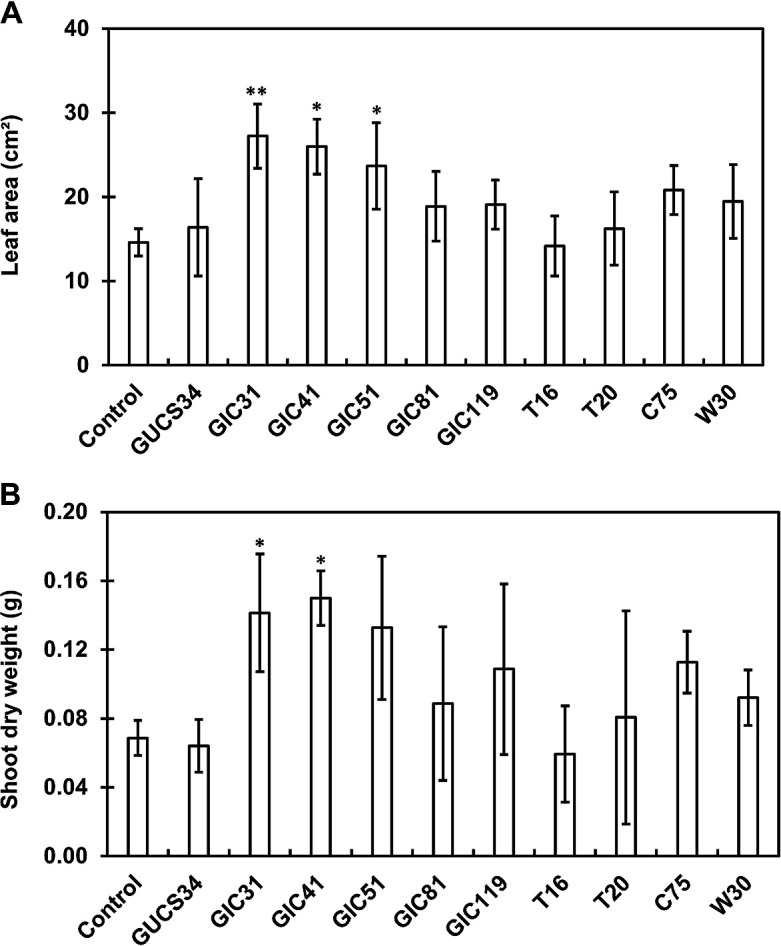
Influence of the *Lysinibacillus* treatment on the leaf area (A) and shoot dry weight (B) of spinach plants grown on Jiffy-7 pellets. Spinach plants were sampled 20 days after treatment. Bars represent the mean±standard deviation of three independent experiments. Significant differences between the control and treatment groups are indicated by asterisks (Dunnett’s test, *P<0.05, **P<0.01).

**Fig. 3.**
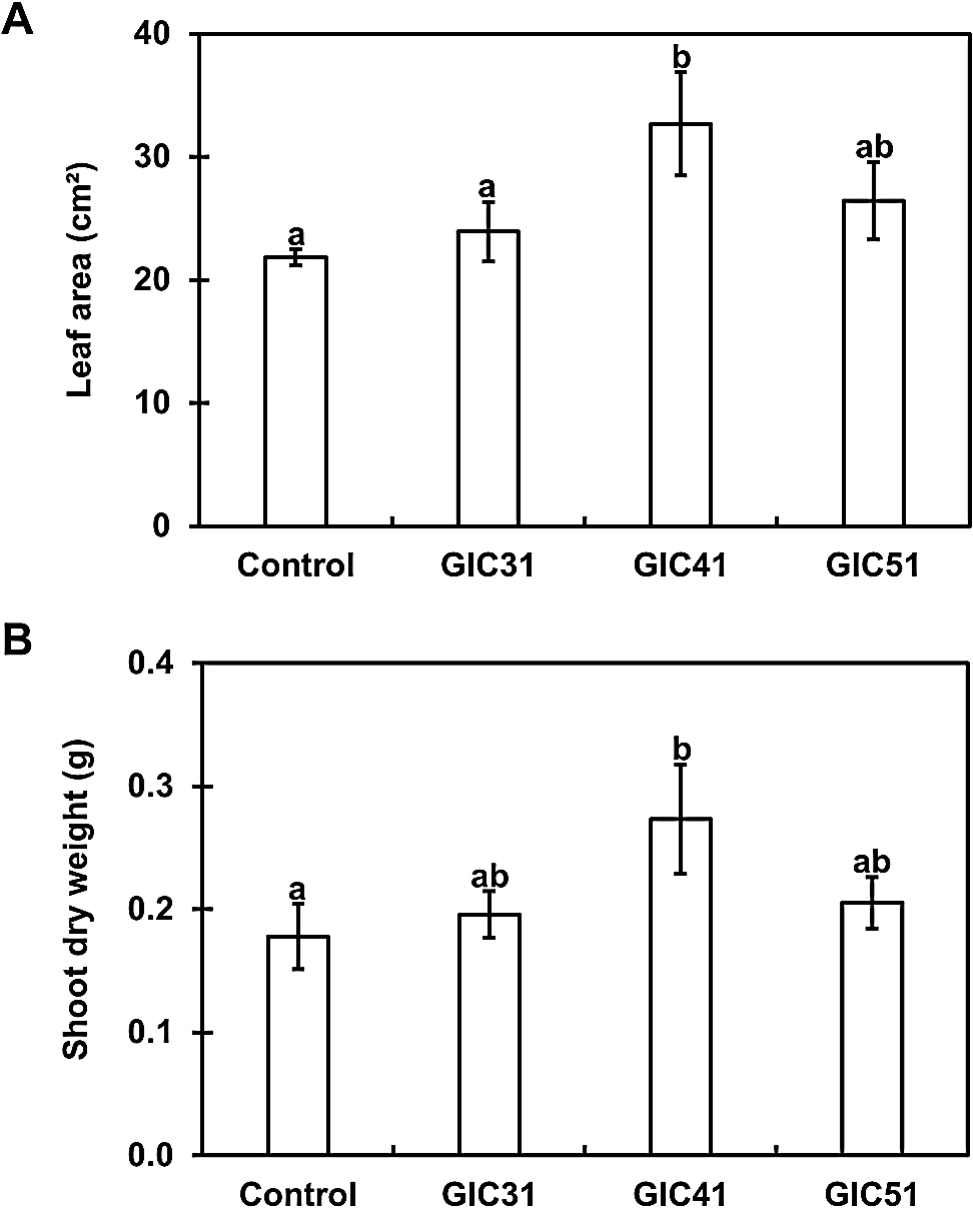
Influence of the *Lysinibacillus* treatment on the leaf area (A) and shoot dry weight (B) of spinach plants grown in pots containing a river sand-vermiculite mixture. Spinach plants were sampled 20 days after treatment. Bars represent the mean±standard deviation of three independent experiments. Significant differences between the treatments are indicated by different letters (Tukey’s multiple comparison of means, *P*<0.05).

Two-step screening was conducted to identify potential *Lysinibacillus* strains that enhance spinach growth. In primary screening, the PGP effects of *Lysinibacillus* strains were evaluated on spinach seedlings grown on Jiffy-7 pellets under controlled conditions. The majority of *Lysinibacillus* strains promoted spinach growth over that of control plants (Fig. 2). Significant enhancements in leaf areas were observed in plants treated with strains GIC31, GIC41, and GIC51. In addition, strains GIC31 and GIC41 significantly increased the dry weights of spinach plants. In secondary screening, the PGP effects of the top three performers in primary screening (strains GIC31, GIC41, and GIC51) were assessed on spinach plants grown in pots containing a river sand–vermiculite mixture that were fertilized once a week under controlled conditions. Only strain GIC41 significantly increased both the leaf area and shoot dry weight (Fig. 3); therefore, this strain was selected for subsequent experiments.

### Efficacy of GIC41 for spinach growth under glasshouse conditions

**Fig. 4.**
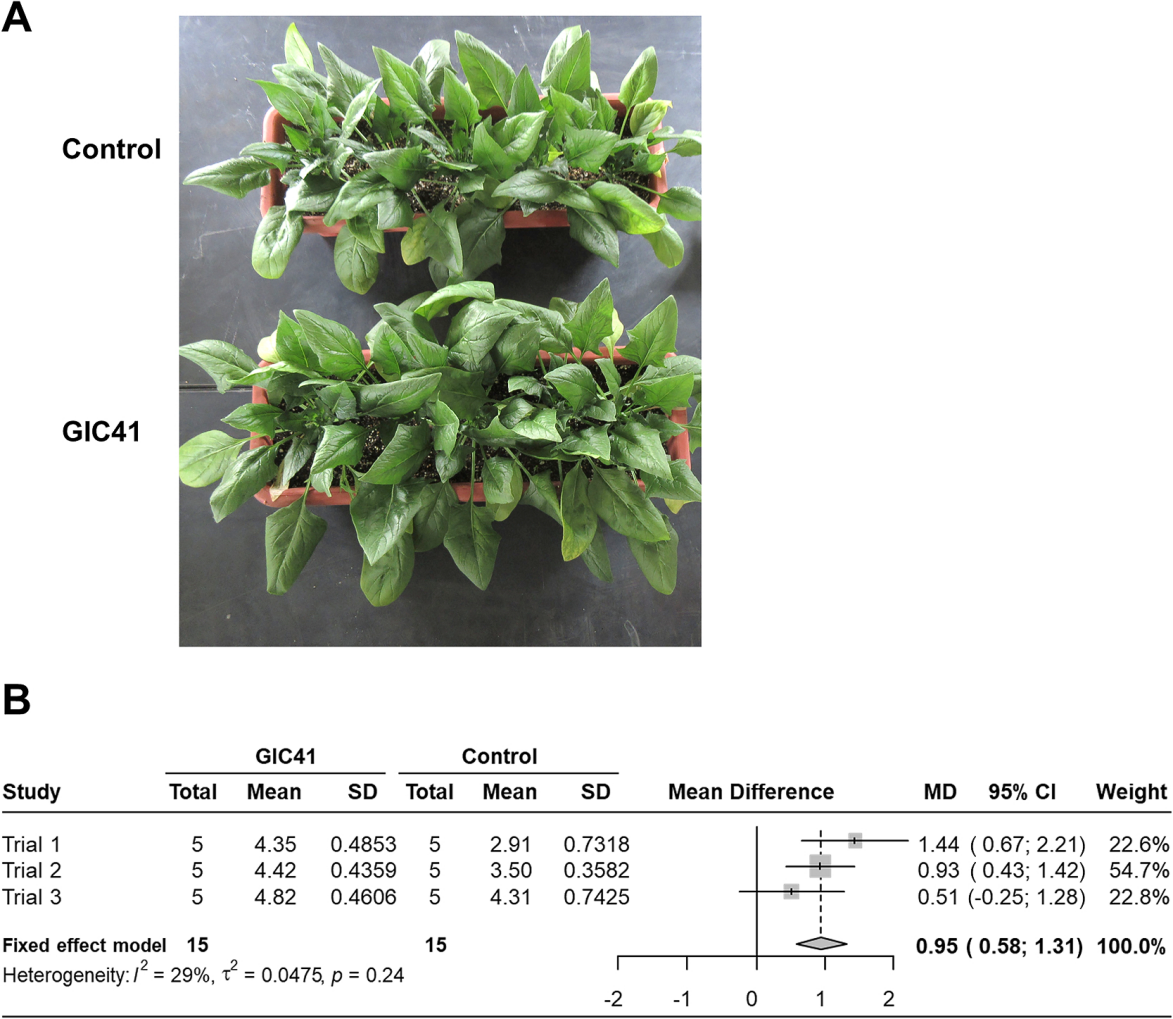
Growth-promoting effects of strain GIC41 on spinach plants under glasshouse conditions. A) Representative examples of control (upper) and GIC41-treated plants (lower) grown for 6‍ ‍weeks. B) A forest plot of a meta-ana­lysis comparing the GIC41 treatment and control treatment for shoot dry weight (g). Shoot dry weight data obtained from three independent trials were analyzed by a meta-ana­lysis. Mean, SD, and MD values represent the mean shoot dry weight (g), standard deviation, and mean difference, respectively. Gray boxes indicate the mean difference for each trial, and horizontal bars show the corresponding 95% confidence interval (95% CI). The diamond indicates pooled mean difference across trials. A fixed effects model was used.

The PGP effects of strain GIC41 on spinach plants grown under glasshouse conditions were evaluated in three independent trials. The results obtained demonstrated that the drench treatment with strain GIC41 on the transplanting day consistently promoted spinach growth across all trials, resulting in an approximate 12–49% increase in the shoot dry weight over that with the control treatment (Fig. 4A and B). A meta-ana­lysis of data from three trials indicated that the mean difference (MD) between the GIC41 and control treatments was 0.95 (95% confidence interval: 0.58–1.31) (Fig. 4B), indicating the significant PGP effects of the GIC41 treatment on the shoot growth of spinach plants under glasshouse conditions.

In contrast, nitrogen and carbon contents (%) in the shoot tissues of GIC41-treated plants were not significantly different from those of control plants (Table 3).

### PGP effects of GIC41 at different fertilization levels

**Fig. 5.**
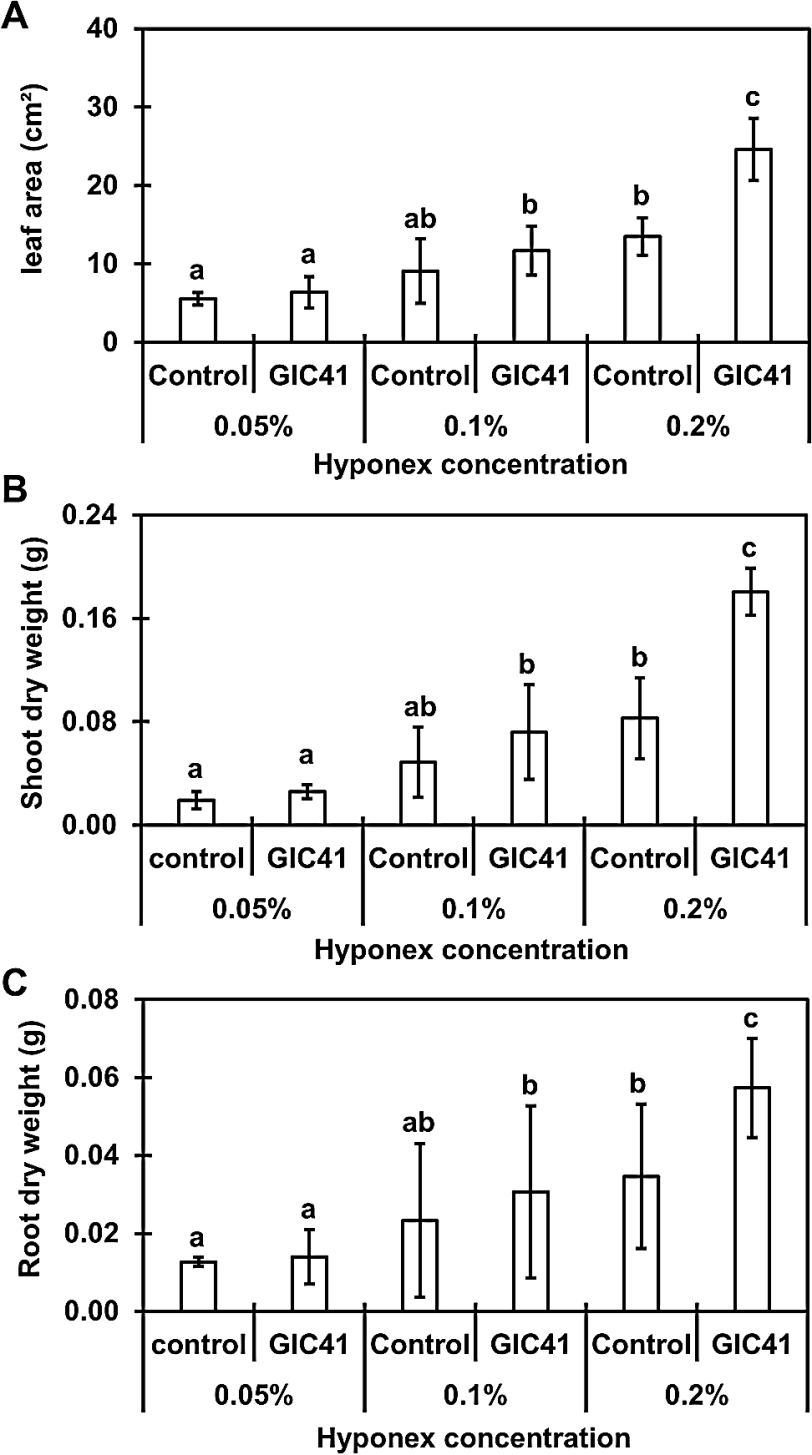
Effects of the GIC41 treatment on the leaf area (A), shoot dry weight (B), and root dry weight (C) of spinach plants fertilized with three different concentrations (0.05, 0.1, and 0.2%) of Hyponex solution. Spinach plants were sampled 20 days after the treatment. Bars represent the mean±standard deviation of three independent experiments. Significant differences between the treatments are indicated by different letters (Tukey’s multiple comparison of means, *P*<0.05).

**Fig. 6.**
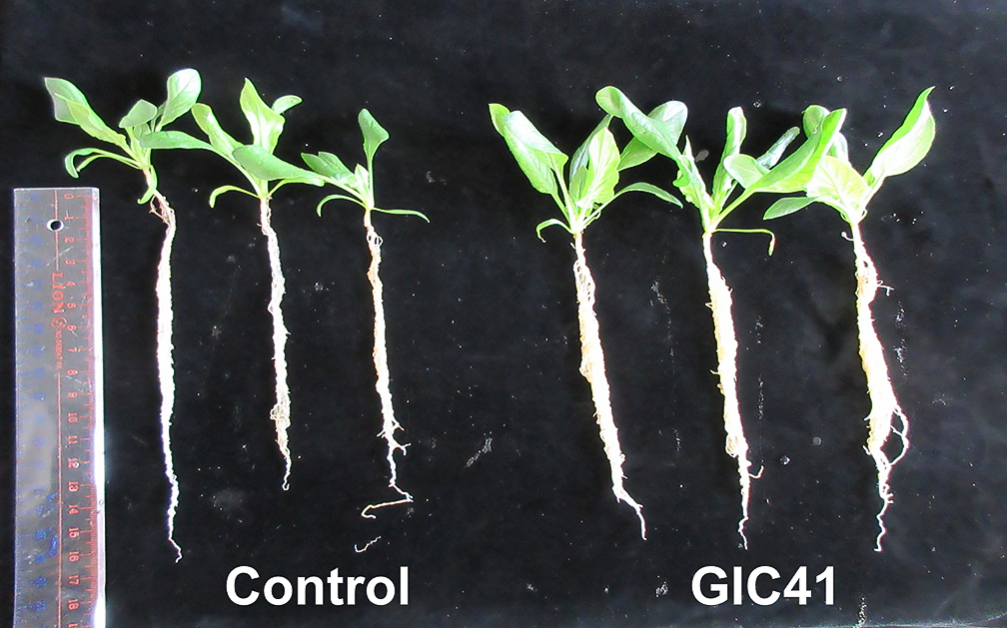
Enhancements induced in lateral root growth by the GIC41 treatment. Spinach plants were fertilized with 0.2% Hyponex solution.

The PGP effects of strain GIC41 were tested on spinach plants fertilized with three different concentrations of Hyponex solution (0.05, 0.1, and 0.2%). The results obtained revealed that irrespective of the fertilizer concentration, the GIC41 treatment promoted shoot and root growth to varying degrees (Fig. 5 and S1). However, significant growth promotion by the GIC41 treatment was only observed in plants that were fertilized with 0.2% (the concentration recommended by the supplier) of Hyponex (Fig. 5 and 6), indicating that strain GIC41 requires a particular level of soil nutrients to fully exert its PGP effects.

## Discussion

The present study aimed to identify *Lysinibacillus* strains with the potential to function as plant biostimulants. We screened 10 *Lysinibacillus* strains, previously isolated from soil and the plant rhizosphere, for their PGP effects on spinach plants. Accordingly, strain GIC41, isolated from paddy field soil, was selected as the best candidate strain because of its consistent performance across screening experiments using spinach seedlings grown in a controlled environmental chamber (Fig. 2). Moreover, the drench treatment with strain GIC41 on the day of transplanting promoted spinach growth and significantly increased shoot biomass 6‍ ‍weeks after transplantation under glasshouse conditions (Fig. 4), indicating the potential of this strain as a plant biostimulant.

Previous studies reported PGP effects following an inoculation with *Lysinibacillus* species, such as *L. sphaericus*, *L. fusiformis*, and *L. chungkukjangi* (Sharma and Saharan, 2015; Yu *et al.*, 2016; Sahu *et al.*, 2018; Borah *et al.*, 2019; Ahsan and Shimizu, 2021). According to the phylogenetic ana­lysis of 16S rRNA gene sequences, strain GIC41 was identified as *L. xylanilyticus* (Fig. 1). The species *L. xylanilyticus* was initially discovered from forest humus as a xylan-degrading bacterium and its validity was confirmed by Lee *et al.* (2010). Since then, several *L. xylanilyticus* strains have been isolated from soil, the rhizosphere, and plant tissues (Esmaeili *et al.*, 2013; Verma *et al.*, 2014; Tan *et al.*, 2015). Moreover, *L. xylanilyticus* strains have been shown to exhibit certain PGP-associated traits, such as IAA production and phosphate solubilization (Verma *et al.*, 2016; De Mandal *et al.*, 2018). However, to the best of our knowledge, its actual capacity to enhance plant growth remains unknown. Therefore, the present study is the first to report the PGP capability of *L. xylanilyticus*.

Many PGPB promote plant growth by increasing the bioavailability of soil nutrients through the solubilization of key mineral nutrients, such as phosphorus and iron (Rawat *et al.*, 2018; Kenneth *et al.*, 2019). However, strain GIC41 lacked the ability to solubilize phosphate or produce siderophores, which are iron-solubilizing agents (Table 2). Furthermore, a simple *in vitro* assay demonstrated that this strain did not exhibit the ability to fix nitrogen, another important trait of many PGPB that promote plant growth (Table 2). Previous studies reported that an inoculation with nitrogen-fixing PGPB increased the plant biomass and also enhanced the nitrogen content in plants (Rokhzadi and Toashih, 2011; Islam *et al.*, 2013). However, an inoculation with GIC41 did not affect the nitrogen content of spinach plants (Table 3), suggesting that the PGP effects of this strain are not attributable to nitrogen fixation.

In comparisons with the control treatment, the GIC41 treatment promoted lateral root development (Fig. 6), which resulted in an increase in the root biomass (Fig. 5C). The lateral roots contribute to nutrient and water absorption from the soil by increasing the overall surface area of the root system (Vessey, 2003). Therefore, enhanced shoot growth in GIC41-treated spinach plants may be attributed to an increase in the uptake of nutrients through the lateral roots. Several PGPR have also been shown to increase the number and/or length of lateral roots, consequently enhancing the growth of the whole plant (Cao *et al.*, 2020; Grover *et al.*, 2021).

Lateral root formation is regulated by the phytohormone auxin and exogenous auxin promotes the production of lateral roots (Fukaki and Tasaka, 2009; Moriwaki *et al.*, 2011; Waidmann *et al.*, 2020). In the present study, *in vitro* assays revealed that strain GIC41 exhibited the ability to produce IAA (Table 2), implying that IAA secreted by this strain plays a key role in the promotion of lateral root growth. The enhancements induced in shoot and root growth by the GIC41 treatment were more prominent under moderately fertilized conditions (fertilized with 0.2% Hyponex solution, the concentration recommended by the supplier) than under less-fertilized conditions (fertilized with 0.1 and 0.05% Hyponex solution) (Fig. 5). This result is consistent with the findings reported by Lamont *et al.* (2014) showing that *Viminaria juncea* inoculated with an IAA-producing *Bacillus megaterium* strain generated a larger root system under moderate nitrogen conditions than under less nitrogen conditions. IAA production by bacteria has also been shown to depend on the availability of nitrogen (Thuler *et al.*, 2003; Tamaki and Mercier, 2007; Shokri and Emtiazi, 2010). Therefore, the soil nutrient status, particularly the content of nitrogen, is a key factor for strain GIC41 to exert its PGP effects.

Although the other *Lysinibacillus* strains examined in the present study were also capable of producing IAA, their PGP effects were inferior to those of strain GIC41, which may be attributed to several factors. Bacteria exhibit different responses to environmental conditions, and these variations affect the production of IAA (Sasirekha *et al.*, 2012; Bharucha *et al.*, 2013; Chandra *et al.*, 2018). Mohite (2013) reported that the quantity of IAA produced by rhizospheric *Bacillus* and *Lactobacillus* strains significantly varied depending on temperature, pH, and types of carbon and nitrogen sources in culture medium. Therefore, environmental conditions, particularly the rhizospheric conditions of spinach plants, may be more favorable for strain GIC41 to synthesize IAA than for the other strains. Another factor is the involvement of PGP substances other than IAA. Among the five major PGP-associated traits (*i.e.*, IAA production, siderophore production, ACC deaminase production, phosphate solubilization, and nitrogen fixation), the *Lysinibacillus* strains examined in the present study only exhibited te ability to produce IAA (Table 2). However, previous studies reported that the production of plant hormones other than IAA, namely, cytokinins and gibberellins, also contributes to the PGP effects of PGPB (Ortíz-Castro *et al.*, 2008; Kang *et al.*, 2014). Moreover, recent studies revealed that volatile organic compounds produced by PGPR activate phytohormone signaling pathways and enhance plant growth (Jishma *et al.*, 2017; Tahir *et al.*, 2017). Therefore, in addition to IAA, strain GIC41 may produce cytokinins, gibberellins, and/or VOCs, and the coordinated effects of these compounds may be responsible for its superior PGP effects. Another possible explanation for this result is differences in the colonization capacities of the strains examined. Successful root colonization is an essential step for PGPR to exert their beneficial effects on plants (Ansari and Ahmad, 2018; Gamez *et al.*, 2019). Although the root-colonizing capacity of *Lysinibacillus* strains was not investigated in the present study, strain GIC41 may have colonized the spinach roots more efficiently than the other strains and continuously secreted IAA into the rhizosphere; therefore, this strain exerted stronger and more stable PGP effects than the other strains. Moreover, previous studies reported that nutrients released from the dead cells of introduced PGPB were taken up by plants and increased their biomass (Macedo-Raygoza *et al.*, 2019; Seerat *et al.*, 2019). Therefore, in addition to the direct growth stimulation via IAA production, a certain proportion of cells may die as GIC41 continues to proliferate around spinach roots, and spinach plants utilize the nutrients released from these dead cells, which results in a significant increase in shoot biomass.

Further studies are needed to elucidate the mechanisms underlying the PGP effects of strain GIC41. We also plan to examine the PGP effects of this strain on various crops in the future.

In conclusion, the results of the present study demonstrated that *L. xylanilyticus* strain GIC41 effectively promoted the growth of spinach plants, and, thus, may contribute to the development of new plant biostimulants.

## Citation

Ahsan, N., Marian, M., Suga, H., and Shimizu, M. (2021) *Lysinibacillus xylanilyticus* Strain GIC41 as a Potential Plant Biostimulant. *Microbes Environ ***36**: ME21047.

https://doi.org/10.1264/jsme2.ME21047

## Supplementary Material

Supplementary Material

## Figures and Tables

**Table 1.** *Lysinibacillus* species identified by a BLAST ana­lysis of nearly full-length 16S rRNA gene sequences

Strain	Origin	% Similarity	GenBank Accession Number
GIC31	Paddy field soil	99.3% with *L. xylanilyticus* DSM 23493	MZ520561
GIC41	Paddy field soil	99.5% with *L. xylanilyticus* DSM 23493	MZ543950
GIC51	Paddy field soil	98.27% with *L. xylanilyticus* DSM 23493	MZ543951
GIC119	Paddy field soil	99.09% with *L. xylanilyticus* DSM 23493	MZ543965
C75	Chinese chive rhizosphere soil	99.58% with *L. xylanilyticus* DSM 23493	MZ543967
GUCS34	Tea garden soil	99.67% with *L. pakistanensis* JCM 18776	MZ543966
T16	Tomato rhizosphere soil	99.25% with *L. capsici* PB300	MZ543969
GIC81	Paddy field soil	100% with *L. capsici* PB300	MZ543964
W30	Welsh onion rhizosphere soil	99.92% with *L. fusiformis* NBRC 15717	MZ543970
T20	Tomato rhizosphere soil	99.67% with *L. fusiformis* NBRC 15717	MZ543972

**Table 2.** *In vitro* plant growth-promoting traits of *Lysinibacillus* strains

Strain	IAA production (μg mL^–1^)	Phosphate solubilization	Siderophore production	ACC deaminase production	Nitrogen fixation
GIC31	1.2	—	—	—	—
GIC41	1.5	—	—	—	—
GIC51	1.0	—	—	—	—
GIC119	1.0	—	—	—	—
C75	2.0	—	—	—	—
GUCS34	1.8	—	—	—	—
T16	1.4	—	—	—	—
GIC81	1.2	—	—	—	—
W30	1.1	—	—	—	—
T20	1.9	—	—	—	—

“—” indicates negative

**Table 3.** Effects of the GIC41 treatment on carbon and nitrogen contents in spinach shoots

Treatment	Carbon content (%)	Nitrogen content (%)
Control	34.5±1.6 a	4.3±1.3 a
GIC41	35.1±1.6 a	3.8±1.1 a

Data represent the mean±standard deviation of three replicates. Means were analyzed for significant differences using the Student’s *t*-test. Values in columns followed by the same letters are not significantly different at *P*<0.05.
