# Supplementary material for: Lysinibacillus xylanilyticus Strain GIC41 as a Potential Plant Biostimulant
Source: Microbes Environ. 2021 Nov 5;36(4):ME21047. doi: 10.1264/jsme2.ME21047 (PMC8674448; doi:10.1264/jsme2.ME21047)
Supplement: Supplementary file 1 — Supplementary Material [file 36_21047_s1.pdf]

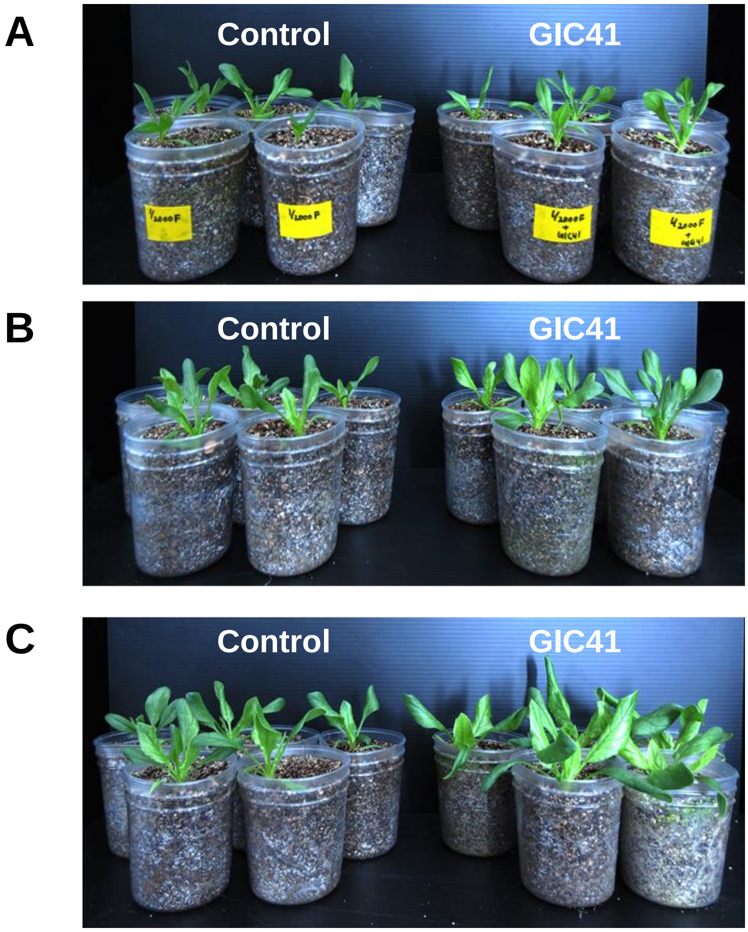

Fig. S1. Effects of the GIC41 treatment on the growth of spinach plants grown on a river sand–vermiculite mixture fertilized with different concentrations (0.05, 0.1, and 0.2%) of Hyponex solution. (A) Spinach plants fertilized with 0.05% Hyponex solution; (B) Spinach plants fertilized with 0.1% Hyponex solution; (C) Spinach plants fertilized with 0.2% Hyponex solution.
